# An Age- Friendly Campus Partnership for Hospitalized Older Adults in the COVID Era and Beyond

**DOI:** 10.1093/geroni/igab046.1497

**Published:** 2021-12-17

**Authors:** Heavner Mojdeh, Marjorie Fass, Christina Cafeo, Giora Netzer, Mangla Gulati, Nicole Brandt, Barbara Resnick, Raya Kheirbek

**Affiliations:** 1 University of Maryland, Baltimore, Maryland, United States; 2 University of Maryland School of Nursing , Baltimore, Baltimore, Maryland, United States; 3 University of Maryland Medical Center, Baltimore, Maryland, United States; 4 University of Maryland School of Pharmacy, University of Maryland School of Pharmacy, Maryland, United States; 5 University of Maryland School of Nursing, Baltimore, Maryland, United States; 6 University of Maryland School of Medicine, University of Maryland School of Medicine, Maryland, United States

## Abstract

As hospitals isolate COVID-19 patients to prevent the spread of this highly contagious disease, patients and family are separated during times of critical illness. For many older adults inflicted with coronavirus it is not the fear of dying that matters the most, it is the fear of dying alone. Utilizing the 4Ms approach, University of Maryland, Baltimore (UMB) and University of Maryland Medical Center (UMMC) responded with several initiatives including intergenerational programs designed to shape and inform the development of future healthcare clinicians in addressing what matters the most to patients and leveraging technology to connect them with families, provide mobility opportunities, monitor medications, and reduce errors.

